# Intermediate and definitive hosts of wild *Schistosoma mansoni*: ecological niche modeling of hosts in low endemicity areas

**DOI:** 10.1590/S1678-9946202466053

**Published:** 2024-09-06

**Authors:** Elisiane Rodrigues dos Santos, Juberlan Silva Garcia

**Affiliations:** 1 Fundação Oswaldo Cruz, Programa de Pós-Graduação em Vigilância e Controle de Vetores, Rio de Janeiro, Rio de Janeiro, Brazil; 2 Secretaria Estadual de Saúde, Núcleo de Vigilância Epidemiológica, Barbacena, Minas Gerais, Brazil; 3 Fundação Oswaldo Cruz, Instituto Oswaldo Cruz, Laboratório de Biologia e Parasitologia de Mamíferos Silvestres Reservatórios, Rio de Janeiro, Rio de Janeiro, Brazil

**Keywords:** Schistosomiasis, Schistosoma mansoni, Ecological niche modeling, Nectomys squamipes

## Abstract

The relationship between the environment and animal life began to be seen as an important tool to help control zoonoses. Climate variations lead to changes in the environment, which can influence the spatial distribution of species and, consequently, the spread of diseases to humans. Considered the main non-human definitive host species of *Schistosoma mansoni* in Brazil, the wild rodent *Nectomys squamipes* plays an important role as a reservoir in maintaining the schistosomiasis cycle in the absence of humans. This study demonstrates the results of ecological niche modeling of intermediate and definitive wild hosts of *S. mansoni* in the Regional Health Superintendence of Barbacena (Minas Gerais State), which has registered 31 municipalities, 80% of which are classified as endemic for parasitosis. Environmental variables associated with the distribution of each species were used based on information from the scientific collections of Global Biodiversity Information Facility (GBIF) and Species Link to project the ecological niche model in the geographic space. Abiotic variables such as the mean annual temperature, isothermality, and precipitation seasonality were obtained from World Clim. Ecological niche modeling of the wild host, *N. squamipes*, revealed the occurrence of the species in geographic overlap with the *Biomphalaria* species. Knowing the influence of bioclimatic variables and identifying favorable conditions for the establishment, occurrence, and distribution of species are important information for developing strategic actions for the surveillance and control of this endemic species. The presence of the definitive wild host needs to be considered by control programs of schistosomiasis.

## INTRODUCTION

Schistosomiasis transmission has been reported in 77 countries, in addition to Brazil. The mollusc species *Biomphalaria glabrata* (Say, 1818), *Biomphalaria tenagophila* (D’orbigny, 1835), and *Biomphalaria straminea* (Dunker, 1848) are involved in the spread of *Schistosoma mansoni* in Brazil, as they were found to be naturally infected^1-3^. In Minas Gerais State, these three species of molluscs of epidemiological importance in the country can be found, contributing to the high prevalence recorded in the state^1^. Schistosomiasis is endemic across a vast area of the territory of Minas Gerais State, with 387 municipalities (45% of the total of 853 municipalities) classified as endemic, distributed in 19 Regional Health Units (RHUs). In total, five municipalities are classified as areas of focus because they are located in RHUs free from schistosomiasis. In the Regional Health Superintendence of Barbacena (21º13’35’’S 43º46’27’’W), of the 31 recorded municipalities, 80% are classified as endemic^4,5^.

Schistosomiasis is a neglected endemic disease related to the deficiency or total absence of basic sanitation^6^. Other conditioning factors contribute to the persistence of the disease, such as the occupation of areas considered at risk for the disease, leisure, professional activities in agriculture and livestock, schooling level, and disinformation^1^. The disease mainly impacts poor and rural communities and contamination occurs via contact with water during agricultural work, domestic activities, or recreational activities. Population movements, such as migration to urban areas and increase in ecotourism in endemic areas, can contribute to the increase in the number of cases and favor the introduction of the disease in new locations^1,2^.

In the 18^th^ century, the relationship between the environment and animal life began to be seen as a factor of great importance for zoonoses^7^. The discovery of several species of rodents, marsupials, and other mammals found naturally infected demonstrated that *S. mansoni* infection was not exclusive to humans. The presence of viable eggs capable of infecting hosts indicates that these species can complete the transmission cycle, thus characterizing their epidemiological importance^7,8^.

Considered the main nonhuman definitive host species of *S. mansoni* in Brazil, the wild rodent *Nectomys squamipes* (Brants, 1827) plays an important role as a reservoir in maintaining the cycle of this parasitic disease in the absence of humans. Its presence in endemic areas is a biological indicator of transmission area and must be considered as one of the factors that hinder control^7,8^.

The species is highly susceptible to infection by different strains of *S. mansoni* and, in general, does not interfere with the animal’s health^9^, not playing a role in regulating the number of rodents in the environment, which favors the transmission of the parasite throughout its lifespan^8^. They do not develop portal hypertension and have a certain immunity, which promotes regulation of the parasite load, despite the ease of reinfection^9^.

Climatic variations can influence the spatial distribution of species and, consequently, the spread of diseases to humans^10^. Ecological niche modeling aims to analyze the conditions that favor establishment of a species in a given geographic space, enabling decision-making in disease prevention actions^10^.

## MATERIALS AND METHODS

The ecological niche model was projected in the geographic space using available data on environmental variables associated with the distribution of each species (*B. glabrata, B. straminea, B. tenagophila*, and *N. squamipes*). The sources of data regarding the biotic factors used were collected from the scientific collections of Global Biodiversity Information Facility (GBIF) and Species Link. Data regarding abiotic factors and bioclimatic variables were collected from World Clim^11^.

To search for GBIF records on the occurrence of species, a filter of points that included geographic coordinates was selected. In Species Link, the filter “geographical coordinates” was selected with the options “original” and “consistent.” The information from both databases was combined and an Excel occurrence spreadsheet was created for each species. At World Clim, a file containing current bioclimatic variables was downloaded.

The R-Studio software (version 4.3.1, R Core Team), a script-based statistical programming environment, was used for ecological niche modeling, in which occurrence data and abiotic variables were loaded. For modeling, the process followed some steps, such as cleaning points of occurrence, removing duplicate records, selecting bioclimatic variables, and plotting and validating models.

The variables associated with the selected species were as follows:

bio_02 = Mean Diurnal Range (Mean of Monthly [max temp – min temp])

bio_03 = Isothermality (P2/P7) (*100)

bio_10 = Mean temperature of the warmest quarter

bio_13 = Precipitation of Wettest Month

bio_15 = Precipitation Seasonality (Coefficient of Variation)

bio_18 = Precipitation of Warmest Quarter

bio_19 = Precipitation of Coldest Quarter

The modeling algorithms used were BIOCLIM and MAXENT (R-Studio packages), which are among the most commonly used tools for ecological niche modeling. They project, in pixels, the area suitable for the establishment of the species, according to the selected bioclimatic variables, and transform the data into binary values on a scale from 0 to 1^12^.

The area under the receiver operating characteristic (ROC) curve (AUC) was used to evaluate the models. This method indicates the model’s discrimination capacity, checking the probability of hits and errors, and correctly interpreting true presences and absences of the model’s points^12^.

## RESULTS

**Figure 1:**
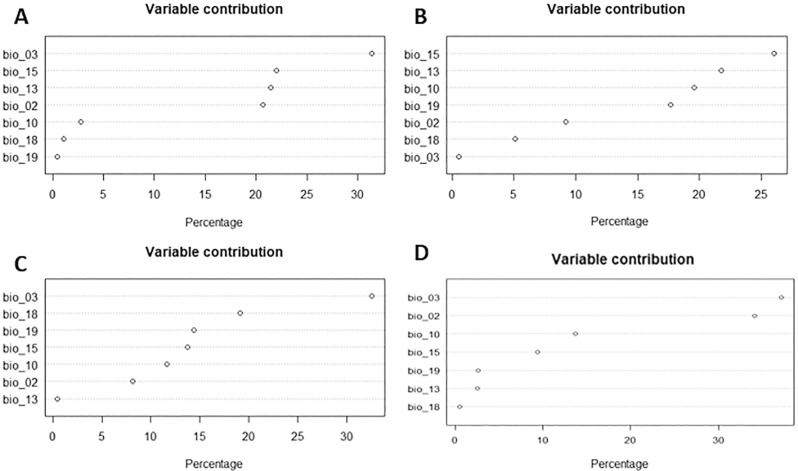
Contribution variables in species distribution modeling: A) *B. glabrata;* B) *B. straminea;* C) *B. tenagophila;* D) *N. squamipes*.

The isothermality bioclimatic variable presented the highest percentage of contribution to the distribution of the species *B. glabrata* and *B. tenagophila* in the environment, and precipitation seasonality was the variable with the greatest contribution in the modeling of the species *B. straminea*. For *N. squamipes*, the isothermality and mean diurnal range variables made the greatest contribution to the modeling (Figure 1).

**Figure 2:**
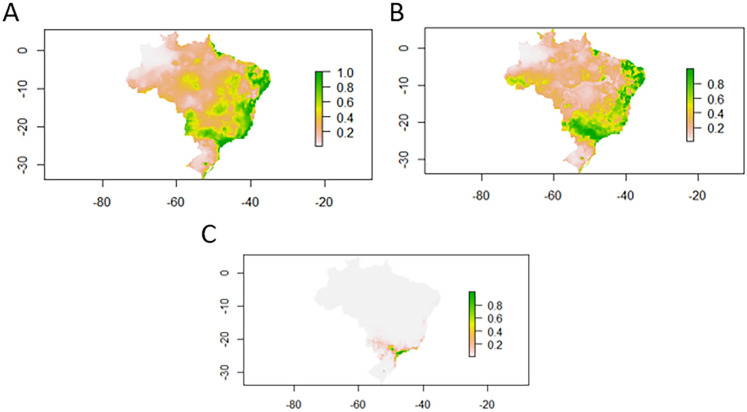
Ecological niche modeling: A) *B. glabrata;* B) *B. straminea;* C) *B. tenagophila*).

The MAXENT algorithm model was built for each species. For *B. glabrata*, 53 points were used in the modeling, presenting higher values and, thus, greater suitability of the species in the studied region (Figure 2A). In modeling the species *B. straminea*, 25 points were used to validate the model. Figure 2B shows a large area with high values for the species, which represents greater suitability in these regions. For *B. tenagophila*, 34 points were used, being the species that presented the smallest area favorable to its establishment, not considered representative for the studied region (Figure 2C).

**Figure 3:**
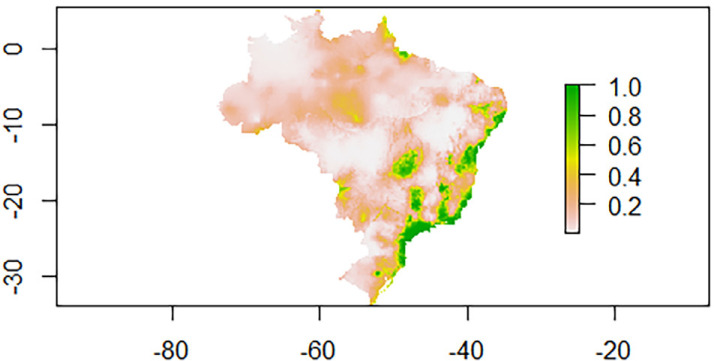
Ecological niche modeling for *N. squamipes*.

In modeling the ecological niche of the wild rodent *N. squamipes*, a wild reservoir, 55 points were used to validate the model. Figure 3 shows the occurrence of the species in geographic overlap with the occurrence of *Biomphalaria* species in the studied region.

Analyzing the AUC—curve generated by MAXENT that determines the validity of the model—for the models, we found that the model for the species *B. tenagophila* presents a value that approaches unity, which means that the model presented high performance. The value (0.8560) means that when two points are drawn, a true presence and a true absence, the algorithm holds an 85% probability of correctly classifying each of them. For the species *B. straminea* (0.6935) and *B. glabrata* (0.5097), despite showing a larger area and representation in the region, the model evaluations hold a 69% and 50% probability of success, respectively. For *N. squamipes*, the model evaluation holds a 58% chance of success (Table 1).

**Table 1:** Results obtained in the evaluation of species models.

Species	Evaluation
	AUC
*Biomphalaria glabrata*	0.5097
*Biomphalaria straminea*	0.6935
*Biomphalaria tenagophila*	0.8560
*Nectomys squamipes*	0.5890

AUC = Area under the curve.

## DISCUSSION

The breeding sites of snail species are influenced by the climate (rainfall, temperature, and humidity), which can determine the establishment and distribution of the species^13^. Climate change, especially the rise in temperatures in the Earth’s atmosphere in the last two centuries, has caused changes in the environment that impact habitats and vectors, consequently influencing disease transmission^14^.

Limnic molluscs can be found in various water collections, such as streams, rivers and lakes, flooded areas, sewage, and drainage ditches, and can also be observed buried at the bottom of the water collection^1,13^. Burying is also a survival mechanism in response to drought of temporary water collections, such as puddles of water formed by rain or floods^1^. Adaptive behaviors of the species allow them to survive even after the use of molluscicides.

Evaluating ecological niche models and the variables that influence species distribution, temperature is a determining factor in the schistosomiasis transmission cycle, as the release of cercariae depends on light and water temperature^15^.

The ideal temperature for the development of molluscs varies from 20 to 26 °C, with *B. glabrata* being the most resistant species to temperature variations. Precipitation is another climate variable that determines the occurrence and increase in the number of *Biomphalaria* breeding sites^15^.

The same variables were used in modeling *N. squamipes*, a semiaquatic species that prefers small tributaries since its swimming capacity is not as efficient^16^. Considering that it uses rivers and streams for movement and dispersion, the characteristic of the region called Campo das Vertentes would be the most favorable to the establishment of the species.

Corroborating the malacological survey on the distribution of *Biomphalaria* species reported in the document “Surveillance and Control of Molluscs of Epidemiological Importance” (SCMEI), based on various databases, publications on schistosomiasis, and information from Brazilian Control Program of Schistosomiasis (PCE)^13^, the ecological niche modeling reveals the occurrence of the three species *B. glabrata, B. straminea*, and *B. tenagophila* in the studied region in varying proportions. The SCMEI reports the occurrence of *Biomphalaria* species in the municipalities referenced by the Regional Health Superintendency (SRS) of Barbacena municipality: (a) *B. glabrata* was recorded in the municipalities of Barbacena, Caranaiba, Catas Altas da Noruega, Conselheiro Lafaiete, and Ouro Branco; (b) *B. tenagophila* was recorded in the municipalities of Barbacena, Conselheiro Lafaiete, and Ouro Branco; and (c) *B. straminea* was recorded in the municipalities of Barbacena, Caranaiba, and Conselheiro Lafaiete^13^.

Ecological niche modeling of the wild host *N. squamipes* revealed the occurrence of the species in geographic overlap with the *Biomphalaria* species, which requires attention in the activities of control programs of schistosomiasis, as the species is responsible for maintaining the cycle of parasitosis in the absence of humans and the region is predominantly classified as endemic.

The correlation between high prevalence of schistosomiasis and poor sanitation infrastructure, including sewage treatment and water supply networks, has been observed in many Brazilian municipalities^17^. Considering the sewage coverage in the studied region, which varies from 28.24% to 90.8% (Brazilian Institute of Geography and Statistics), and the coverage of water supply networks, from 40.94% to 96.75%^18^, we can correlate the maintenance of schistosomiasis transmission due to environmental and socioeconomic factors. The snail, an intermediate host of the disease, in water collections, associated with precarious basic sanitation conditions and the exposure of communities in domestic, professional, and/or activities are important factors for the persistence of transmission^1^.

## CONCLUSION

We identified areas with the presence of intermediate and definitive host species involved in the schistosomiasis transmission cycle via ecological niche modeling. The presence of these species associated with poor coverage of sewage systems and water supply networks are determining factors for the maintenance of disease transmission. Knowing the influence of bioclimatic variables and identifying the conditions that favor the establishment, occurrence, and distribution of the species is important information for developing strategic actions for the surveillance and control of endemic diseases.

Understanding the concept of One Health, which highlights the relationship between human, animal, and environmental health, is crucial for combating the disease. Therefore, continuous efforts are needed to understand the factors that complicate disease control and resist conventional interventions.
